# Differential roles of breakfast only (one meal per day) and a bigger breakfast with a small dinner (two meals per day) in mice fed a high-fat diet with regard to induced obesity and lipid metabolism

**DOI:** 10.1186/1740-3391-10-4

**Published:** 2012-05-15

**Authors:** Yuta Fuse, Akiko Hirao, Hiroaki Kuroda, Makiko Otsuka, Yu Tahara, Shigenobu Shibata

**Affiliations:** 1 Laboratory of Physiology and Pharmacology, School of Advanced Science and Engineering, Waseda University, Wakamatsu-cho 2-2, Shinjuku-ku, Tokyo, 162-8480, Japan

**Keywords:** Meal timing, Circadian rhythm, Metabolic syndrome, Breakfast versus dinner, High-fat diet

## Abstract

**Background:**

Recent studies on humans and rodents have suggested that the timing of food intake plays an important role in circadian regulation and metabolic health. Consumption of high-fat foods during the inactive period or at the end of the awake period results in weight gain and metabolic syndrome in rodents. However, the distinct effects of breakfast size and the breakfast/dinner size ratio on metabolic health have not yet been fully examined in mice.

**Methods:**

We examined whether the parameters of metabolic syndrome were differentially affected in mice that consumed a large meal at the beginning of the awake period (breakfast; one meal group) and a relatively smaller meal at end of the awake period (dinner; two meals group). The mice of each group were provided equal food volume per day.

**Results:**

Mice on one meal exhibited an increase in body weight gain, hyperinsulinemia, hyperleptinemia, and a decrease of gene expression associated with β-oxidation in adipose tissue and liver compared with those on two meals. The circadian expression pattern of the *Clock* gene in mice on one meal was disturbed compared with those on two meals.

**Conclusions:**

In conclusion, a bigger breakfast with a smaller dinner (two meals per day) but not breakfast only (one meal per day) helps control body weight and fat accumulation in mice on a high-fat meals schedule. The findings of this study suggest that dietary recommendations for weight reduction and/or maintenance should include information on the timing and quantity of dietary intake.

## Background

Excess caloric intake is associated with the development of obesity, increased adiposity, glucose intolerance, insulin resistance and hyperleptinemia. A number of recent studies in animals have linked energy regulation and the circadian clock at the molecular, physiological, and behavioral levels [1]. *Clock* gene mutant mice exhibit obesity and hyper-cholesterol under conditions of high-fat and high-cholesterol diets, respectively [2,3]. In addition, *Clock* mutant mice exhibit abnormal activity and feeding rhythms; they eat not only during night period but also during the day period. These mutant mice also show abnormal *Clock* gene expression in peripheral organs [2,3]. High-fat diet-induced [4,5] and genetically obese mice, such as KK-Ay and db/db mice [6,7], show abnormal circadian behavior and *Clock* gene expression in peripheral organs.

Recent papers on humans and rodents have suggested that feeding habits (timing of food intake) play an important role in circadian regulation and metabolic health. In human studies, eating meals irregularly was positively associated with metabolic syndrome in a population-based, cross-sectional study [8]. In addition, human studies on non-breakfast eaters and night-eating syndrome patients are consistent with the timing of food intake being a determining factor for weight gain [9,10]. Arble et al [11] reported that limiting high-fat intake to the 12-h light phase (sleep period for rodents) results in significantly greater weight gain in mice compared with mice restricted to high-fat feeding during the 12-h dark phase (awake period for rodents). In a restricted feeding experiment, food restricted to the normal sleep period increased the body weight of rats [12]. Although, these rodent experiments are important, limiting food availability to the time in which these nocturnal animals are normally sleeping is associated with disturbed biological rhythms. Recently, Bray et al [13] examined how manipulating the timing of feeding during the normal awake period influences metabolic syndrome. They demonstrated that the time of day at which carbohydrate versus fat is consumed markedly influences multiple cardiometabolic syndrome parameters and that consumption of a high-fat diet at the end of the awake period leads to increased weight gain, adiposity, glucose tolerance and hyperinsulinemia. Recently, Wu et al., [14] reported that rats given two meals per day during the latter half of the active period exhibit high adipose tissue accumulation compared with those given three meals per day during active periods or with those given two meals per day during the earlier half of the active period under the same amount of total food every day. Thus, the absence of breakfast or dinner significantly affects body weight gain and adipose tissue accumulation.

In humans, regular consumption of breakfast is associated with a healthier body weight compared with skipping breakfast [15]. Similarly, children who regularly eat breakfast tend to have a lower body-mass-index and are less likely to be overweight than those who eat breakfast less frequently [15]. Such findings suggest that eating breakfast is a good lifestyle habit for maintaining good health. If skipping breakfast in humans [15] and rodents [14] is a bad habit with regard to obesity, then in contrast, consuming a larger breakfast may be a good habit. Therefore, we investigated which of the following options is beneficial for maintaining normal weight and preventing excess adipose tissue accumulation: 1) breakfast only (one meal); or bigger breakfast/smaller dinner (2 meals).

Peripheral circadian clock systems are entrained by a light–dark cycle through the suprachiasmatic nucleus, which is the main oscillator, and also by daily restricted feeding [16]. The phase of the liver clock has been reported to be dependent on meal size and meal timing under a two meals per day schedule and also on nutritional composition of the meal [17,18]. Thus, eating habits may contribute to not only body weight gain, but also the circadian pattern of *Clock* gene expression in peripheral organs.

In the present study therefore, we examined whether the parameters of metabolic syndrome were differentially affected in mice that consumed a large meal at the beginning of the awake period (breakfast) and a relatively smaller meal at end of the awake period (dinner), compared with mice that ate an only breakfast. Experiments were conducted using a high-fat diet as it facilitated obesity under the free-feeding (FF) condition, making it is easy to distinguish the effect of meal pattern differences on obesity. As parameters of metabolic syndrome, we measured the serum levels of insulin, leptin, and adiponectin, because insulin and leptin levels are increased while adiponectin level is decreased in obesity and diabetes [19,20]. Many enzymes involved in fatty acid and triacylglycerol synthesis are tightly regulated during fasting and feeding [21]. Sterol regulatory element-binding protein-1c (SREBP-1c) plays a central role in transcription regulation of genes for hepatic lipid synthesis, and Srebp-1c mRNA levels are regulated by the fasting/feeding cycle, high intake of nutrients such as carbohydrate and fat, and the circadian clock [22]. In addition, a critical role of SREBP-1c in the transcriptional activation of lipogenic genes such as a fatty acid synthase (FAS) has been shown [23]. FABP1 and MTP contribute to the import of fatty acids and the export of very low-density lipoprotein (VLDL), respectively [24,25]. PPARα and CPT1 play critical roles in beta oxidation in the mitochondria [26]. In the present study, we measured expression levels of *Srebp-1c, Fas, Fabp1, Mtp, Pparα,* and *Cpt1* in liver and white adipose tissue, because these genes contribute to lipid metabolism.

## Methods

### Animals

Six-week-old male ICR mice (Tokyo Laboratory Animals, Tokyo, Japan) were housed at a temperature of 22 ± 2°C, a humidity of 60 ± 5%, and under a 12 h light/12 h dark (LD) cycle (lights on from 08:00 to 20:00). Zeitgeber time 0 (ZT0) was designated as lights-on time and ZT12 as lights-off time under the LD cycle. The mice were fed a normal commercial diet (Catalog # MF; Oriental Yeast Co. Ltd., Tokyo, Japan) and provided water *ad libitum* before the experiment. Experimental animal care was conducted with permission from the Committee for Animal Experimentation of the School of Science and Engineering at Waseda University (permission # 09A11, 10A11) and in accordance with the Law (No. 105) and Notification (No. 6) of the Japanese Government.

### High-fat diet

**Figure 1:**
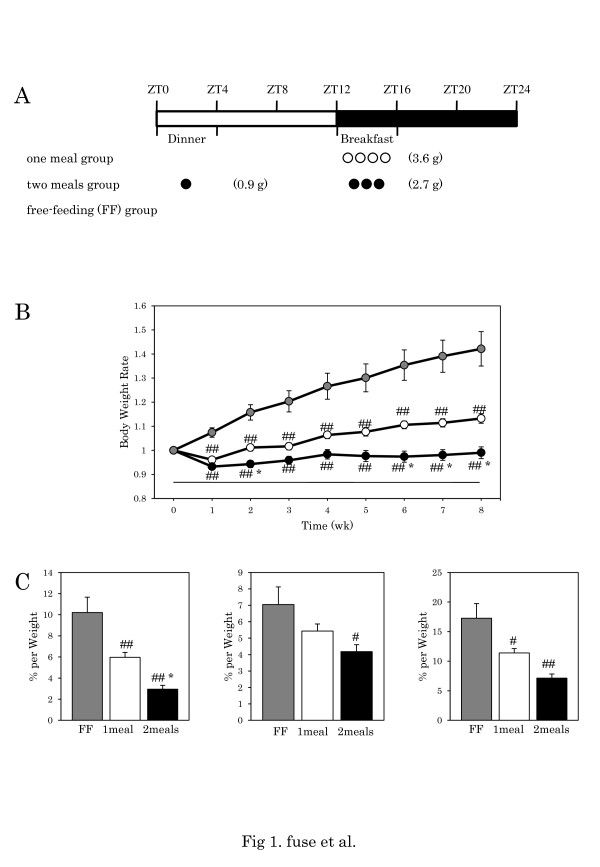
**Body weight gain and quantity of adipose tissue.A**: Experimental schedule. Eight-week-old mice were divided into three groups as follows: 3.6 g of high-fat chow at ZT12 as breakfast (one meal group); and 2.7 g of high-fat chow at ZT12 as breakfast and 0.9 g at ZT0 as dinner (two meals group), and a free-feeding (FF) group. White circle: one meal; black circle: two meals. **B**: Increase in body weight. Body weight at the start of restricted feeding is designated as 1. Restricted feeding starts at 0 (X-axis). Gray circle: FF; white circle: one meal; black circle: two meals. Data are presented as mean ± SEM values (FF, n = 16; one meal, n = 20; two meals, n = 19). # p < 0.05, ## p < 0.01 vs. FF, * p < 0.05, vs. one meal (Tukey-Kramer test). **C**: Visceral fat, subcutaneous fat and total body fat. Y-axis: (adipose tissue weight/body weight) × 100 (%). Gray column: FF; white column: one meal; black column: two meals. Data are presented as mean ± SEM values (FF, n = 16; one meal, n = 20; two meals, n = 19). # p < 0.05, ## p < 0.01 vs. FF, * p < 0.05, ** p < 0.01 vs. one meal (Tukey-Kramer test).

A high-fat diet (15% beef tallow (wt/wt) added to a standard diet; Funabashi Farm Co., Ltd.) was fed to the mice. The high-fat diet comprised 40% calories from fat, while the normal diet comprised 10% calories from fat. Feeding volume under the FF condition of the high-fat diet was approximately 4.5 g per day. We decided to give 3.6 g (80% FF) per day to avoid saving the diet until the next meal time. Eight-week-old mice were divided into three groups (3.6 g of high-fat diet at ZT12 (one meal group), 2.7 g of high-fat diet at ZT12 and 0.9 g at ZT0 (two meals group), and the FF group) (Figure 1A). This feeding schedule was implemented for 8 weeks, and body weight was measured weekly.

### Collection of blood and tissue for hormones and gene expression assay

Mice were maintained on the feeding schedule for 8 weeks before sacrifice. After measurement of body weight, mice were fasted for 24 h, and 4–6 mice were sacrificed at each time point (ZT0, ZT6, ZT12, and ZT18). A terminal blood sample was collected from the retroorbital sinus under deep ether anesthesia. Blood samples then were allowed to clot. The clots were removed, and samples were centrifuged at 4°C for 30 min at 3300 × g. Serum aliquots were aspirated and stored (750 to 1000 μl) in sealable polypropylene microcentrifuge tubes at −80°C for subsequent analysis. Visceral and subcutaneous adipose were dissected and weighed, and liver tissue was also dissected from each mouse. After measurement of hormone levels and mRNA levels at each time point, mean values (FF, n = 16; one meal, n = 20; and two meals, n = 19) along with ZTs (4–6 mice for each ZT) were calculated. Mean values were calculated from average levels throughout the day.

### Serum insulin, leptin, adiponectin assay

Plasma concentrations of insulin (Shibayagi Co., Ltd., Tokyo, Japan) and leptin and adiponectin (R&D Systems, Inc., Cosmo Bio Co., Ltd., Tokyo Japan) in mice were determined in duplicate by enzyme-linked immunosorbent assay (ELISA) kits, according to the manufacturer’s protocol. Detectability of the assay was within the calibration limits, and the intra-assay coefficient of variation was <10% for insulin, leptin, and adiponectin.

### RNA isolation and real-time RT-PCR

Pieces of adipose and liver tissue were dissolved into ISOGEN Reagent (Nippon Gene, Tokyo, Japan), and total RNA was isolated. A 50-ng aliquot of total RNA was reverse transcribed and amplified using the One-Step SYBR RT-PCR Kit (TaKaRa, Otsu, Japan) in the iCycler (BIO RAD, Hercules, CA). Specific primer sequences (Table 1) were designed using Primer3 Input (version 0.4.0, Steve et al., 2000), and these primers contain introns in each gene. RT-PCR was executed under the following conditions: cDNA synthesis at 42°C for 15 min followed by 95°C for 2 min, PCR amplification for 40 cycles with denaturation at 95°C for 5 s, and annealing and extension at 60°C for 20 s. The conditions of real-time reverse transcription polymerase chain reaction (RT-PCR) were the same as those of our previous paper [18]. The relative light unit of the PCR product of the target gene was normalized to that of *Gapdh*. Data were analyzed by the delta-delta Ct method. Melt curve analysis was performed to check for nonspecific products. Results indicated no amplification of nonspecific products*.*

**Table 1:** Primer sequences of each gene

** Gene name**	**Forward**	**Reverse**
*Gapdh*	TGGTGAAGGTCGGTGTGAAC	AATGAAGGGGTCGTTGATGG
*Per2*	TGTGTGCTTACACGGGTGTCCTA	ACGTTTGGTTTGCGCATGAA
*Bmal1*	CTAATTCTCAGGGATGTGACCG	AACAAGCTCTGGCCAATAAGG
*Srebp-1c*	CGCTACCGGTCTTCTATCAATG	CAAGAAGCGGATGTAGTCGATG
*Fas*	GGCAGAGAAGAAAGCTGTGG	TCGGATGCCTAGGATGTGTG
*Fabp1*	GCAGAGCCAGGAGAACTTTG	TGATGTCCTTCCCTTTCTGG
*Mtp*	GCCCTAGTCAGGAAGCTGTG	CCAGCAGGTACATTGTGGTG
*Pparα*	TCTTCACGATGCTGTCCTCCT	GGAACTCGCCTGTGATAAAGC
*Cpt1*	GTGACTGGTGGGAGGAATAC	GAGCATCTCCATGGCGTAG

### Statistical analysis

Data are expressed as mean ± SEM values. For statistical analysis, a one-way or two-way ANOVA was applied in StatView software (SAS Institute, Cary, NC), and *post hoc* analysis was conducted using a Tukey-Kramer test. A *p* value of 0.05 or less was considered statistically significant.

## Results

### Body weight gain and adipose weight

When we started the experiments, body weight was not significantly different between the three groups (36.7 g for one meal, 36.8 g for two meals, and 36.1 g for FF) (Figure 1A, 1B). We decided to give 3.6 g (80% FF) of high-fat diet per day to avoid saving excess food until the next meal time. Eight-week-old male mice were divided into two groups as follows: 3.6 g of high-fat diet at ZT12 (one meal group), and 2.7 g of high-fat diet at ZT12 and 0.9 g at ZT0 (two meals group) (Figure 1A). This feeding schedule was implemented for 8 weeks, and body weight was measured weekly. The time course of body weight gain was evaluated using a one-way ANOVA, and differences between the groups were assessed by the two-way ANOVA (Figure 1B). There were significant differences between the one meal and two meals groups (F = 3.9, p < 0.001, Two-way ANOVA) and between the two meals and FF groups (F = 9.7, p < 0.001, Two-way ANOVA) (Figure 1B). The two meals group exhibited significantly less weight gain than the one meal group at weeks 2, 6, 7, and 8 (p < 0.05, Tukey-Kramer test). There was also a large increase of visceral adipose tissue in the high-fat FF mice (4.2% for normal fat; 10.2% for high-fat), and there was a lower percentage of visceral fat in the two meals group than in the one meal group (p < 0.05, Tukey-Kramer test) (Figure 1C).

### Serum insulin, leptin, and adiponectin

**Figure 2:**
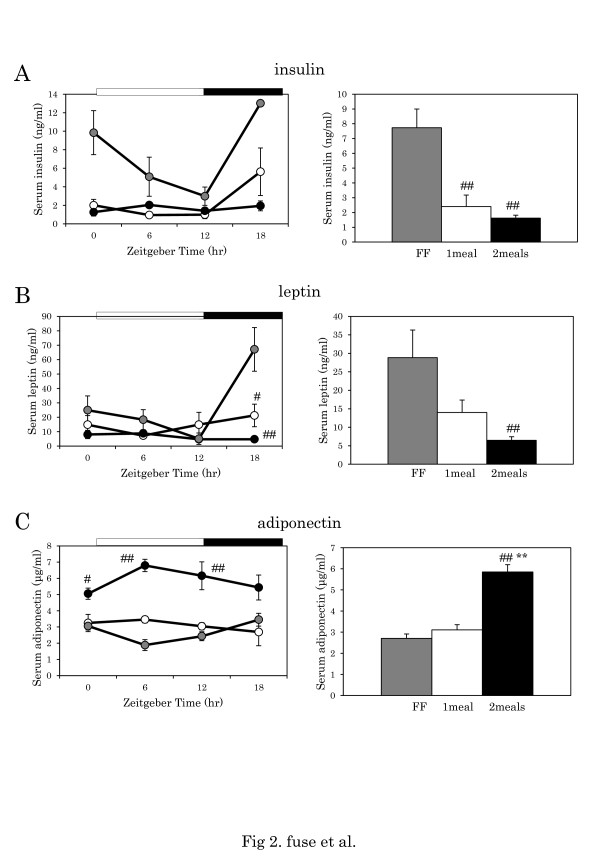
**Serum levels of insulin, leptin and adiponectin.A**: Daily pattern of insulin level. **B**: Daily pattern of leptin level. **C**: Daily pattern of adiponectin level. Horizontal open and closed bars indicate light and dark periods, respectively. The number of mice at each time point was 4–6 for each group. Columns in the right panels show the average level throughout the day. Data are presented as mean ± SEM (FF, n = 16; one meal, n = 20; two meals, n = 19). Gray circle and column: FF; white circle and column: one meal; black circle and column: two meals. ## p < 0.01 vs. FF, ** p < 0.01 vs. one meal (Tukey-Kramer test).

The purpose of measuring the change in levels of insulin, leptin, and adiponectin vs. mean levels is that serum insulin and leptin have a daily rhythm [5]. After 8 weeks of the one meal or two meals per day schedule, mice for each time point were sacrificed after 24 h of fasting. The daily significant rhythms of insulin, leptin, and adiponectin were observed in the high-fat group and FF groups (Table 2, one-way ANOVA). On the other hand, one-way ANOVA showed no significant daily rhythms of insulin leptin and adiponectin in the one meal or two meals groups (Table 2, one-way ANOVA). Two-way ANOVA revealed significant differences in insulin, leptin, and adiponectin between the FF group and two meals group (Table 2). Although serum insulin levels were significantly higher in the FF group than in the one meal and two meals groups (p < 0.01, respectively, Tukey-Kramer test), no significant difference in daily average levels was shown between the one meal and two meals groups (Figure 2A). Average serum leptin levels were significantly lower in the two meals group than in the FF group (p < 0.01, Tukey-Kramer test) (Figure 2B). Average serum adiponectin levels were significantly higher in the two meals group than in the one meal and FF groups (p < 0.01, Tukey-Kramer test) (Figure 2C).

**Table 2:** Analysis of serum insulin, leptin, and adiponectin levels by one-way and two-way ANOVA

	**One-way**			**2-way**		
	**FF**	**1meal**	**2meals**	**FF vs. 1meal**	**FF vs.2meals**	**1meal vs.2meals**
Insulin	F = 7.5, p < 0.01	F = 2.7, p > 0.05	F = 0.9, p > 0.05	F = 1.7, p > 0.05	F = 8.1, p < 0.01	F = 2.5, p > 0.05
Leptin	F = 7.3, p < 0.01	F = 0.7, p > 0.05	F = 1.3, p > 0.05	F = 3.1, p < 0.05	F = 8.6, p < 0.01	F = 1.5, p > 0.05
Adiponectin	F = 4.7, p < 0.05	F = 0.4, p > 0.05	F = 1.2, p > 0.05	F = 2.5, p > 0.05	F = 3.0, p < 0.05	F = 0.6, p > 0.05

### Daily rhythms of *clock* gene expression in visceral adipose and liver tissue

**Figure 3:**
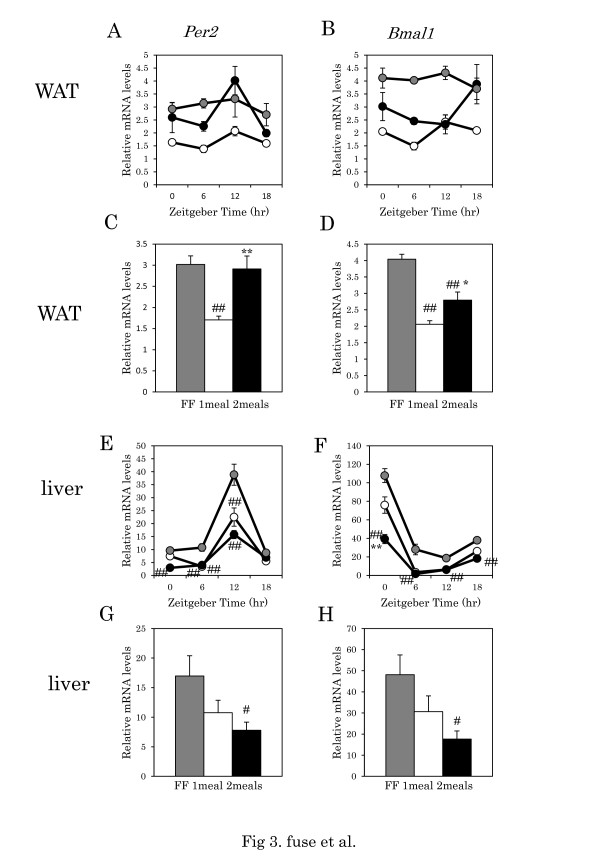
**Clock gene expression in visceral adipose (WAT) and liver tissues in mice with one meal or two meals.A**, **B**: Daily pattern of *Per2* and *Bmal1* mRNA level in WAT, respectively. **E**, **F**: Daily pattern of *Per2* and *Bmal1* mRNA level in liver, respectively. The number of mice at each time point was 4–6 for each group. **C**, **D**, **G**, **H**: Columns show the average level of gene expression throughout the day. Data are presented as mean ± SEM values (FF, n = 16; one meal, n = 20; two meals, n = 19). Y-axis: the relative levels of each data set are normalized to the corresponding *Gapdh* mRNA levels. Gray circle and column: FF; white circle and column: one meal; black circle and column: two meals. ## p < 0.01, #p < 0.05 vs. FF, * p < 0.05, ** p < 0.01 vs. one meal (Tukey-Kramer test).

The daily rhythm of *Per2* and *Bmal1* gene expression in adipose tissue was rhythmic in the one meal and two meals groups, but not in the FF group (Table 3, one-way ANOVA) (Figure 3A, 3B). Two-way ANOVA revealed no significant differences between FF group and the one meal group, between FF and the two meals group, or between the one-meal and two meals group in daily rhythm of *Per2* and *Bmal1* gene expression (Table 3) (Figure 3A, 3B). Daily amplitude of *Per2* and *Bmal1* gene expression rhythm evaluated by top level/bottom level was 1.2 and 1.2 for the FF group, 1.5 and 1.6 for the one meal group, and 2.0 and 2.0 for the two meals group, respectively (Figure 3A, 3B).

**Table 3:** Analysis of liver and WAT clock gene expression by one-way and two-way ANOVA

	**One-way**			**2-way**		
	**FF**	**1meal**	**2meals**	**FF vs. 1meal**	**FF vs.2meals**	**1meal vs.2meals**
WAT Bmal1	F = 0.7, p > 0.05	F = 4.1, p < 0.05	F = 4.3, p < 0.05	F = 1.2, p > 0.05	F = 2.7, p > 0.05	F = 2.9, p > 0.05
WAT Per2	F = 0.4, p > 0.05	F = 4.5, p < 0.05	F = 3.8, p < 0.05	F = 0.4, p > 0.05	F = 1.3, p > 0.05	F = 2.0, p > 0.05
liver Bmal1	F = 61.5, p < 0.001	F = 40.6, p < 0.001	F = 23.7, p < 0.001	F = 1.6, p > 0.05	F = 17.9, p < 0.001	F = 7.4, p < 0.001
liver Per2	F = 39.7, p < 0.001	F = 15.2, p < 0.001	F = 24.2, p < 0.001	F = 4.0, p < 0.05	F = 14.2, p < 0.001	F = 2.1, p > 0.05

The daily rhythm of *Per2* and *Bmal1* gene expression in liver was rhythmic in all three groups (Table 3, one-way ANOVA) (Figure 3E, 3D). Expression of the *Bmal1* gene in the two meals group was significantly lower than that in the one meal or FF groups (Table 3, two-way ANOVA) (Figure 3E, 3D). The daily amplitude of *Per2* and *Bmal1* gene expression rhythm evaluated by top level/bottom level was 4.5 and 5.8 for the FF group, 6.6 and 22.0 for the one meal group, and 5.4 and 24.4 for the two meals group, respectively (Figure 3E, 3D).

### Daily rhythms of clock-controlled adipogenesis gene expression in visceral adipose and liver tissue

**Figure 4:**
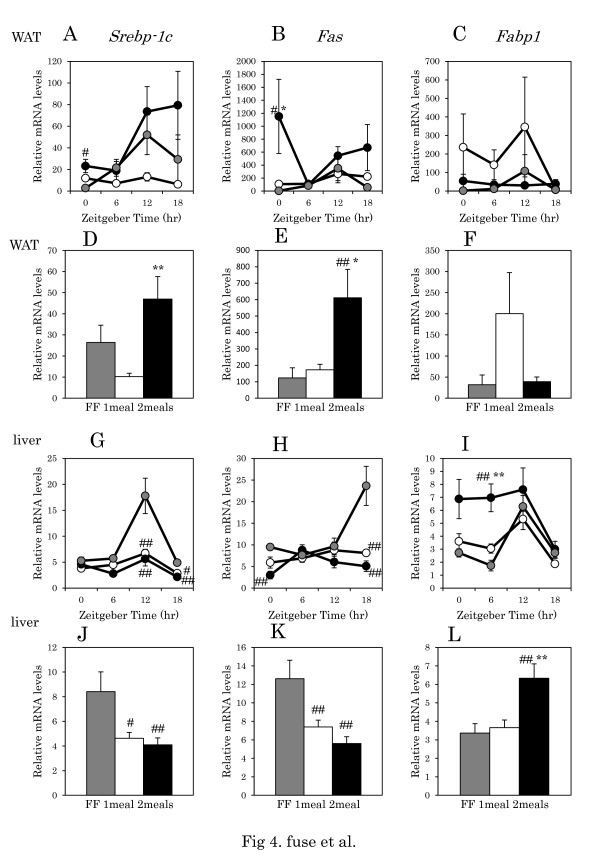
**Metabolic related gene expression in (WAT and liver tissues in mice with one meal or two meals.A**, **B**, **C**: Daily pattern of *Srebp-1c*, *Fas,* and *Fabp1* mRNA level in WAT, respectively. **G**, **H**, **I**: Daily pattern of *Srebp-1c*, *Fas,* and *Fabp1* mRNA level in liver, respectively. The number of mice at each time point was 4–6 for each group. **D**, **E**, **F**, **J**, **K**, **L**: Columns show the average level of gene expression throughout the day. Data are presented as mean ± SEM values (FF, n = 16; one meal, n = 20; two meals, n = 19). Y-axis: the relative levels of each data set are normalized to the corresponding *Gapdh* mRNA levels. Gray circle and column: FF; white circle and column: one meal; black circle and column: two meals. ## p < 0.01, #p < 0.05 vs. FF, * p < 0.05, ** p < 0.01 vs. one meal (Tukey-Kramer test).

**Figure 5:**
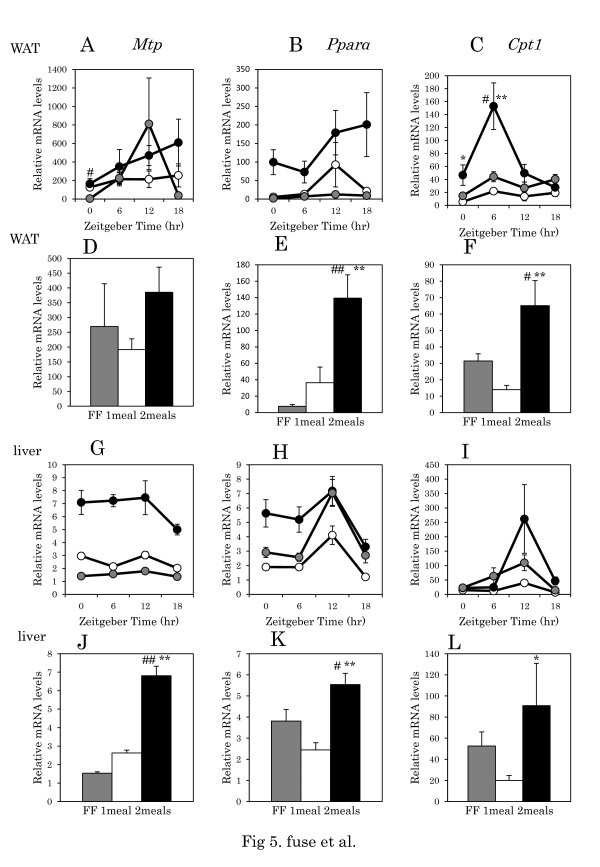
**β-oxidation related gene expression in WAT and liver tissues in mice with one meal or two meals.A**, **B**, **C**: Daily pattern of *Mtp*, *Pparα,* and *Cpt1* mRNA levels in WAT, respectively. **G**, **H**, **I**: Daily pattern of *Mtp*, *Pparα,* and *Cpt1* mRNA level in liver, respectively. **D**, **E**, **F**, **J**, **K**, **L**: Columns show the average level of gene expression throughout the day. Data are presented as mean ± SEM values (FF, n = 16; one meal, n = 20; two meals, n = 19). Y-axis: the relative levels of each data set are normalized to the corresponding *Gapdh* mRNA levels. The number of mice at each time point was 4–6 for each group. Gray circle and column: FF; white circle and column: one meal; black circle and column: two meals. ## p < 0.01, #p < 0.05 vs. FF, * p < 0.05, ** p < 0.01 vs. one meal (Tukey-Kramer test).

There were no significant differences in daily rhythms of mRNA levels of adipose *Srebp-1c*, *Fas*, *Fabp1, Mtp, Ppara,* and *Cpt1*within the one meal, two meals, and FF groups (Table 4, one-way ANOVA) (Figures 4A, 4B, 4C, 5A, 5B, 5C). There were also no significant difference in *Srebp-1c*, *Fas*, *Fabp1, Mtp,* and *Ppara* between the one meals vs FF groups, two meals vs FF groups, and one meal vs two meals groups (Table 4, two-way ANOVA), except for *Cpt1* between the FF vs two meals groups, and between the one meals vs two meals groups. The average *Srebp-1c* expression in the two meals group was significantly higher than in the one meal group (p < 0.01, Tukey-Kramer test) (Figure 4D). The average *Fas* expression was also significantly higher in the two meals groups than in the one meal and FF groups (p < 0.05, p < 0.01 respectively, Tukey-Kramer test) (Figure 4E). There was no significant difference in the average *Fabp1* and *Mtp* expression (Figure 4F, 5D). The average *Pparα* expression was significantly higher in the two meals group than in the one meal and FF groups (p < 0.01 respectively, Tukey-Kramer test) (Figure 5E). The average *Cpt1* expression was significantly higher in the two meals group than in the one meal and FF groups (p < 0.01, p < 0.05 respectively, Tukey-Kramer test) (Figure 5F).

**Table 4:** Analysis of liver and WAT metabolism-related gene expression by one-way and two-way ANOVA

	**One-way**			**2-way**		
	**FF**	**1meal**	**2meals**	**FF vs. 1meal**	**FF vs.2meals**	**1meal vs.2meals**
WAT Srebp-1c	F = 1.8, p > 0.05	F = 0.7, p > 0.05	F = 2.7, p > 0.05	F = 2.1, p > 0.05	F = 0.5, p > 0.05	F = 2.8, p > 0.05
WAT Fas	F = 1.9, p > 0.05	F = 0.9, p > 0.05	F = 1.7, p > 0.05	F = 0.7, p > 0.05	F = 2.0, p > 0.05	F = 2.0, p > 0.05
WAT Fabp1	F = 1.3, p > 0.05	F = 0.4, p > 0.05	F = 0.2, p > 0.05	F = 0.2, p > 0.05	F = 1.4, p > 0.05	F = 0.4, p > 0.05
WAT Mtp	F = 2.2, p > 0.05	F = 0.5, p > 0.05	F = 1.2, p > 0.05	F = 2.2, p > 0.05	F = 1.4, p > 0.05	F = 0.6, p > 0.05
WAT Ppara	F = 1.0, p > 0.05	F = 1.4, p > 0.05	F = 1.1, p > 0.05	F = 0.8, p > 0.05	F = 0.9, p > 0.05	F = 0.5, p > 0.05
WAT Cpt1	F = 3.5, p > 0.05	F = 2.3, p > 0.05	F = 2.8, p > 0.05	F = 0.6, p > 0.05	F = 4.8, p < 0.01	F = 6.5, p < 0.01
liver Srebp-1c	F = 12.3, p < 0.001	F = 5.1, p < 0.05	F = 2.8, p > 0.05	F = 7.5, p < 0.001	F = 7.8, p < 0.001	F = 0.8, p > 0.05
liver Fas	F = 8.9, p < 0.01	F = 0.8, p > 0.05	F = 3.6, p < 0.05	F = 6.0, p < 0.01	F = 9.6, p < 0.001	F = 1.3, p > 0.05
liver Fabp1	F = 13.7, p < 0.001	F = 5.1, p < 0.05	F = 1.8, p > 0.05	F = 1.8, p > 0.05	F = 1.7, p > 0.05	F = 0.5, p > 0.05
liver Mtp	F = 1.6, p > 0.05	F = 4.3, p < 0.05	F = 1.1, p > 0.05	F = 1.7, p > 0.05	F = 0.6, p > 0.05	F = 0.6, p > 0.05
liver Ppara	F = 13.9, p < 0.001	F = 9.2, p < 0.01	F = 2.8, p > 0.05	F = 2.0, p > 0.05	F = 1.3, p > 0.05	F = 0.8, p > 0.05
liver Cpt1	F = 4.5, p < 0.05	F = 4.0, p < 0.05	F = 2.9, p > 0.05	F = 2.5, p > 0.05	F = 1.1, p > 0.05	F = 2.4, p > 0.05

Daily rhythms mRNA levels of liver *Srebp-1c*, *Fas*, *Fabp1, Mtp, Ppara,* and *Cpt1* showed significant differences in the FF group, except for *Mtp,* and in the one meal group, except for *Fas* (Table 4, one-way ANOVA). There are no significant differences in gene expression in liver between the one meal vs. two meals groups (Table 4, two-way ANOVA). There were no significant differences in average *Srebp-1c* and *Fas* expressions between the one meal and two meals groups (Figure 4J, 4K). The average *Fabp1* expression was significantly higher in the two meals group than in the one meal and FF groups (p < 0.01 respectively, Tukey-Kramer test) (Figure 4L). The average *Mtp, Pparα, and Cpt1* expression was significantly higher in the two meals group than in the one meal group (p < 0.01, p < 0.05 respectively, Tukey-Kramer test) (Figure 5J, 5K, 5L).

## Discussion

To understand the influence of time-of-day-dependent meal size on metabolic syndrome, we examined whether a bigger breakfast/smaller dinner ratio or breakfast only during the normal awake period under a one or two high-fat meals per day schedule can affect metabolic syndrome parameters in mice. At first, restricted feeding (80% of FF, one meal, or two meals per day) of high-fat diets significantly attenuated the metabolic syndrome. Body weight gain and body fat were significantly higher in the high-fat FF group compared with the restricted-feeding group. Thus, the experiments demonstrate that restricted feeding (80% of FF volume) is remarkably effective for protecting against obesity and metabolic syndrome. In the present experiments, a high-fat diet was given to control mice under free-feeding conditions. An intake strategy of 80% (FF in terms of meal patterning or a 3–6 meal/day approach) for the control group may be suitable for an ideal meal patterning comparison while still being isocaloric. Therefore, such a feeding schedule may yield detailed differences of meal patterning.

We found that body weight gain is significantly increased in mice with breakfast only compared with mice with a bigger breakfast/smaller dinner ratio. Therefore, it is suggested that two meals per day rather than one meal such as breakfast only is a preferable eating schedule for protecting against obesity and metabolic syndrome produced by the high-fat diet. In the present study, we analyzed the subcutaneous, visceral, and total fat ratio in three groups of mice because visceral and subcutaneous fat is associated with metabolic syndrome (leptin and adiponectin secretion, and insulin resistance) in humans [19,20]. It is also known that visceral adipose percentage, insulin levels, and leptin levels are higher in persons with more severe metabolic disorder [19,20].

To understand the mechanism mediating differences of body weight gain between groups, we examined the daily rhythm patterns of serum levels of insulin, leptin, and adiponectin, as well as their average levels throughout the day. Significantly daily rhythms of serum insulin, leptin, and adiponectin were observed in the high-fat FF group but not in the one meal or two meals groups. Therefore, we cannot explain the difference of body weight gain in relation to the difference in daily rhythm of these hormones. Insulin and leptin levels were significantly lower, and adiponectin levels were higher in the two meals per day group than in the FF group, suggesting that restricted feeding with two meals a day is remarkably effective for protecting against obesity and metabolic syndrome. Insulin and leptin levels were relatively higher in the one meal group compared with the two meals group. Thus, parameters of metabolic syndrome, such as visceral adipose percentage, hyperinsulinemia, and hyperleptinemia may partially explain why mice with breakfast only show more severe metabolic syndrome than those with a bigger breakfast/ smaller dinner. We observed hyperinsulinemia in the FF group, but not in the one meal or two meals groups. Therefore, insulin sensitivity to meal patterns through multiple post-meal blood glucose sampling should be measured in future experiments.

Average expression levels of liver *Srebp-1c* and *Fas* were higher in the FF group, and expression levels of these genes were lower in the two meals group (p < 0.01) than in the one meal group (p < 0.05). Thus, lower expression levels of these genes may contribute to the lower metabolic syndrome seen in the two meals group, because FAS is a key enzyme in the fatty acid synthetic pathway [23] and because mRNA levels of *Fas* in liver of obese mice fed a high-fat diet are extremely high [5]. FABP1 and MTP are known to contribute to the import of fatty acids and the export of VLDL, respectively [24,25]. In the present study, the two meals group exhibited high expression levels of liver *Fabp1* and *Mtp* gene expression than the one meal or FF groups, suggesting that the metabolic circulation of fatty acid through the liver may be facilitated in the two meals group. Gene expression of *Ppara* and *Cpt1* occurs in liver and adipose tissue in the two meals group, but not in the one meal or FF groups. As both genes play critical roles in beta oxidation in the mitochondria [26], up-regulation observed in the two meals group suggests a high rate of fatty acid consumption. These mechanisms may underlie the small adipose accumulation and low weight gain in the two meals group.

Recently, Bray et al [13] reported that mice fed a high-fat diet at the beginning of the awake period retain metabolic flexibility in response to dietary challenges later in the awake period, and conversely, that consumption of a high-fat diet at the end of the awake period leads to increased weight gain, adiposity, glucose intolerance, hyperinsulinemia, hypertriglyceridemia, and hyperleptinemia. Similarly, Wu et al., [14] recently reported that rats given two meals per day during the latter half of the active period exhibited high adipose tissue accumulation than those given two meals per day during the earlier half of the active period or those given three meals per day during active periods with the same amount of total food per day. The concordance of Wu’s data with Bray’s data clearly suggests that rodents fed a high-fat diet or a high-calorie diet at the beginning of the awake period may be in better health than mice fed a high-fat diet at the end of the awake period or a high-calorie diet. In addition, our results suggest that breakfast only (one meal) is worse for heath than a big breakfast with a small dinner (two meals). Here we adopted a feeding schedule with a breakfast/dinner ratio of 3:1 because we wanted to know the effect of a bigger breakfast. In future however, we should examine the effect of other breakfast/dinner ratios such as 2:2, 1:3, or 0:4.

In the present study, we found arrhythmicity of *Per2* and *Bmal1* gene expression in the white adipose tissues of mice with FF, but rhythmicity of these genes was retained in the one- or two–meal schedules. Daily amplitude of *Per2* and *Bmal1* gene expression evaluated by top level/bottom level was higher in the two meals group (2.0 and 2.0) than in the one meal (1.5 and 1.6) or FF groups (1.2 and 1.2). Thus, the expression pattern of the *Clock* gene in mice with two meals is similar to that in mice with FF of normal diet [27]. In a recent paper we demonstrated that the phase of *Clock* gene expression in liver is dependent on the food volume eaten at each mealtime when mice are given one meal or two meals per day [18]. Animal models of disrupted circadian rhythm, such as *Clock* mutant mice [2] and mice housed in low levels of light at night [28], as well as shift-work animals [12], exhibit increased weight gain and hypertriglyceridemia. Arble et al [11] reported that restricted feeding with a high-fat diet during the sleep period is associated with increased weight gain in mice. Such restrictive food intake at this inappropriate time of the day has been shown to desynchronize the peripheral clock. Several papers demonstrate the existence of numerous lipid metabolism-related clock-controlled genes, including *Srebp-1c* and *Fas,*[5,29-31]. Circadian rhythm disorder of *Clock* gene expression seen in the FF and one meal groups may disturb the normal expression of clock-controlled genes associated with digestive metabolism. In animal studies, obesity observed in KK-Ay mice and db/db mice and feeding of a high-fat diet was found to attenuate the circadian expression of *Clock* genes [4-7].

The parameters of feeding rhythm and eating pattern are size, frequency of meals, and meal timing. In human experiments, obese children consume less energy at breakfast, miss breakfast more frequently, and consume a higher percentage of energy at dinner than adults [32]. A decline in the frequency of eating breakfast and the emergence of the obesity epidemic have raised scientific interest in the possible causal role of breakfast in weight control and related disease risks [33]. In the present study, mice were given a high-fat diet from 8 weeks old until 16 weeks old. Therefore, in future experiments, a high-fat diet should be given to mice just after weaning to understand dietary habits in adolescents.

In conclusion, a bigger breakfast with a smaller dinner (two meals per day) but not breakfast only (one meal per day) helps control body weight and fat in mice on a high-fat meals schedule. The findings of this study suggest that dietary recommendations for weight reduction and/or maintenance should include information on the timing and quantity of dietary intake.

## Competing interests

The authors declare that they have no competing interests.

## Authors’ contributions

YF and AH designed the experiments, collected data, and wrote the manuscript. HK, MO, and YT participated in the design of this study and analyzed data. SS managed the laboratory and participated in the analysis and discussion of the results. All authors read and approved the manuscript.
